# Deciphering Pro-angiogenic Transcription Factor Profiles in Hypoxic Human Endothelial Cells by Combined Bioinformatics and *in vitro* Modeling

**DOI:** 10.3389/fcvm.2022.877450

**Published:** 2022-06-17

**Authors:** Arne Schmidt, Maximilian Fuchs, Stevan D. Stojanović, Chunguang Liang, Kevin Schmidt, Mira Jung, Ke Xiao, Jan Weusthoff, Annette Just, Angelika Pfanne, Jörg H. W. Distler, Thomas Dandekar, Jan Fiedler, Thomas Thum, Meik Kunz

**Affiliations:** ^1^Institute of Molecular and Translational Therapeutic Strategies (IMTTS), Hannover Medical School, Hanover, Germany; ^2^Fraunhofer Cluster of Excellence Immune-Mediated Diseases (CIMD), Hanover, Germany; ^3^Fraunhofer Institute for Toxicology and Experimental Medicine (ITEM), Hanover, Germany; ^4^Department of Cardiology and Angiology, Hannover Medical School, Hanover, Germany; ^5^Department of Bioinformatics, Biocenter, University of Würzburg, Würzburg, Germany; ^6^Department of Internal Medicine 3 – Rheumatology and Immunology, Universitätsklinikum Erlangen, Friedrich-Alexander University (FAU) of Erlangen-Nürnberg, Erlangen, Germany; ^7^Chair of Medical Informatics, Friedrich-Alexander University (FAU) of Erlangen-Nürnberg, Erlangen, Germany

**Keywords:** hypoxia, transcription factor, endothelial, angiogenesis, promoter profiling, signaling

## Abstract

**Background:**

Constant supply of oxygen is crucial for multicellular tissue homeostasis and energy metabolism in cardiac tissue. As a first response to acute hypoxia, endothelial cells (ECs) promote recruitment and adherence of immune cells to the dysbalanced EC barrier by releasing inflammatory mediators and growth factors, whereas chronic hypoxia leads to the activation of a transcription factor (TF) battery, that potently induces expression of growth factors and cytokines including platelet-derived growth factor (PDGF) and vascular endothelial growth factor (VEGF). We report a hypoxia-minded, targeted bioinformatics approach aiming to identify and validate TFs that regulate angiogenic signaling.

**Results:**

A comprehensive RNA-Seq dataset derived from human ECs subjected to normoxic or hypoxic conditions was selected to identify significantly regulated genes based on (i) fold change (normoxia vs. hypoxia) and (ii) relative abundancy. Transcriptional regulation of this gene set was confirmed *via* qPCR in validation experiments where HUVECs were subjected to hypoxic conditions for 24 h. Screening the promoter and upstream regulatory elements of these genes identified two TFs, KLF5 and SP1, both with a potential binding site within these regions of selected target genes. *In vitro*, siRNA experiments confirmed SP1- and KLF5-mediated regulation of identified hypoxia-sensitive endothelial genes. Next to angiogenic signaling, we also validated the impact of TFs on inflammatory signaling, both key events in hypoxic sensing. Both TFs impacted on inflammatory signaling since endogenous repression led to increased NF-κB signaling. Additionally, SP1 silencing eventuated decreased angiogenic properties in terms of proliferation and tube formation.

**Conclusion:**

By detailed *in silico* analysis of promoter region and upstream regulatory elements for a list of hypoxia-sensitive genes, our bioinformatics approach identified putative binding sites for TFs of SP or KLF family *in vitro*. This strategy helped to identify TFs functionally involved in human angiogenic signaling and therefore serves as a base for identifying novel RNA-based drug entities in a therapeutic setting of vascularization.

## Introduction

Constant supply of oxygen is crucial for cellular and tissue homeostasis regulating energy metabolism (1). In contrast, hypoxia is defined as the state that describes an impaired oxygen partial pressure loss in blood and tissue, eventually leading to a shortage of oxygen and thus detrimental for organ function. Besides physiological oxygen levels, also the oxygen concentration at which endogenous hypoxia gene responses are activated vary between different tissues and developmental stages (1). Of note, hypoxia itself can occur naturally, for example at high altitudes (2) or in a variety of disease models such as stroke (3), cardiovascular disease (4), and cancer (5). Cellular or tissue hypoxia is known to regulate gene expression on multiple levels: at DNA level transcription factors (TFs) are present, which are activated under hypoxic conditions (e.g., HIF-1α), and additionally the activity of histone-modifying enzymes that modulate chromosomal organization is oxygen-dependent (6, 7). This complex molecular interplay is resulting in dynamic chromatin structures leading to active or repressed genomic sites as shown in earlier studies investigating angiogenic hypoxic gene signature (8). Hypoxic signaling is causally linked to inflammation as hypoxia-inducible factor 1 (HIF-1α) stimulates the expression of NF-κB, which subsequently triggers the expression of inflammatory genes (9). In a feedback-loop manner, NF-κB signaling was also shown to transcriptionally activate HIF-1α and basal NF-κB activity was demonstrated to be required for HIF-1α protein accumulation under hypoxia (10). Furthermore, hypoxic stress stimulates the induction of autophagy in order to maintain cellular survival (11, 12). In cancer, the reciprocal crosstalk between NF-κB and autophagy can either repress or promote tumorigenesis. Here, stimulus and context determine the fate of tumorigenesis (13). Taken together, these information link hypoxia with autophagy, innate immunity and inflammation *via* multi-lateral interactions. ECs form the inner layer of blood vessels and thus act a vital part in a variety of biological processes such as angiogenesis (14), inflammation (15), and nutrient supply *via* the circulatory system (16). The response of ECs to changes in oxygen availability crucially depends on how the underlying condition manifests in a time-dependent manner. As a first response to acute hypoxia, ECs promote recruitment and adherence of immune cells to the dysbalanced endothelium by expressing and releasing inflammatory mediators and growth factors, whereas long-term hypoxia causes the activation of a TF battery (e.g., HIF-1α), that potently induces expression of growth factors and cytokines including platelet-derived growth factor (PDGF) and vascular endothelial growth factor (VEGF) (17). Abundant expression of those cytokines initiates proliferation and migration of ECs (14) which ultimately supports the formation of fresh blood vessels in poorly perfused tissue areas. Understanding the molecular pathways of angiogenic gene patterns can thus highlight novel therapeutic approaches in the aforementioned organ disorders.

Herein, we apply a hypoxia-minded, targeted bioinformatics promoter screening approach followed by experimental validation of hypoxia-sensitive endothelial genes regulated by the TFs SP1 and KLF5 including functional hypoxia-regulated events. Our approach is not limited to a specific biological setting, thus serves as a general base for identifying novel drug entities either supporting or inhibiting these networks in a therapeutic setting.

## Results

### Bioinformatics Screening Identified SP1 and KLF5 as Potential Drivers of Endothelial Cell Transcriptional Response to Hypoxia

**FIGURE 1:**
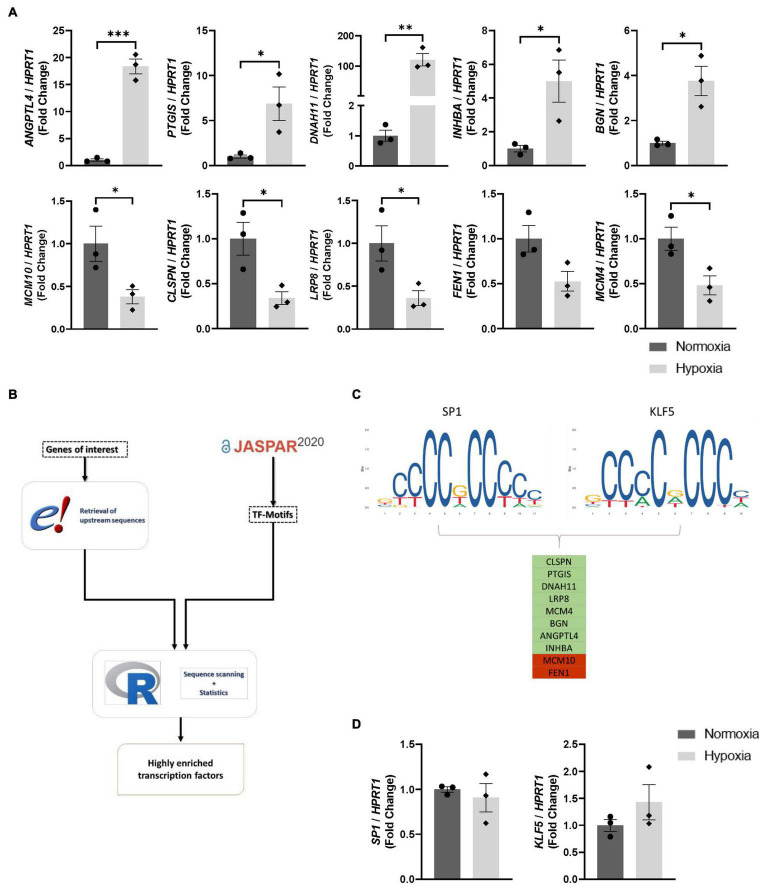
Transcription factors SP1 and KLF5 are potential regulators of EC transcriptional response to hypoxia. **(A)** mRNA levels of selected genes from a previously reported RNA-Seq dataset were validated in HUVECs after 24 h of hypoxia *via* qPCR (*n* = 3). **(B)** Bioinformatic pipeline for the selection of transcription factors. **(C)** *In silico* prediction of potential binding sites for SP1 and KLF5 in hypoxia-sensitive geneset. **(D)** qPCR data of *SP1* and *KLF5* mRNA levels in HUVECs after 24 h of hypoxia (*n* = 3). Each dot resembles the mean value of all technical replicates from an independent experiment. *p ≤ 0.05, ^**^p ≤ 0.01, ^***^p ≤ 0.001.

Initially, a previously reported RNA-Seq dataset (GSE70335) derived from human ECs subjected to normoxic or hypoxic conditions (8) was selected to identify significantly regulated genes based on (i) fold change (normoxia vs. hypoxia) and (ii) relative abundancy. Transcriptional regulation of this gene set was confirmed *in vitro via* qPCR in validation experiments. In line with the experimental setup of the reported RNA-Seq dataset, HUVECs were incubated for 24 h at low oxygen conditions to validate differential gene expression in qPCR experiments (Figure 1A). Combining upstream regulatory elements of this gene set with TF binding matrices in our efficient analysis pipeline allowed high throughput screening of our gene set for multiple TF binding sites (Figure 1B). Screening the promoter and upstream regulatory elements of genes, whose dysregulation could be validated in qPCR experiments, identified the TFs KLF5 and SP1 harboring a possible binding site within these regions in 8 out of the 10 differentially expressed genes. As the top two TFs in our results they were selected for further validation experiments (Figure 1C). All results from the bioinformatics analysis can be found in Supplementary Material (Supplementary Table 1). Given this prediction, we examined if hypoxia regulates endogenous SP1 and KLF5 expression in ECs by monitoring their mRNA levels after various periods of hypoxia. Both, *SP1* as well as *KLF5* mRNA levels remained constant after hypoxia treatment for 24 h (Figure 1D). In line, time lapse hypoxia experiments in HUVECs revealed that mRNA levels of both, *SP1* and *KLF5* remain constant during the first 24 h, whereas after 48 h, mRNA levels are significantly elevated compared to normoxic cells (Supplementary Figure 1). Thus, we hypothesize that mainly SP1 and KLF5 localization but not their expression levels act as a potential driver of hypoxic gene expression in human ECs *in vitro*.

### SP1 and KLF5-Knockdown Mediated Differential Gene Expression After Hypoxia

**FIGURE 2:**
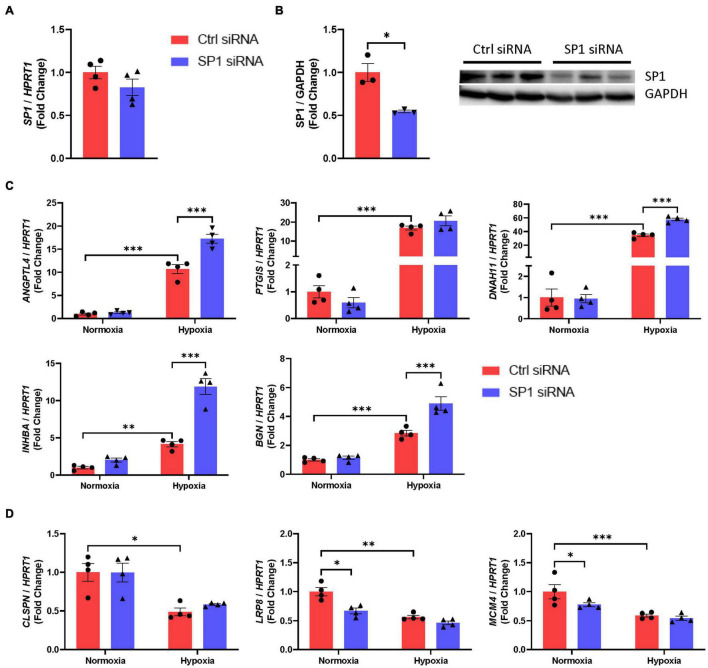
SP1 differentially regulates hypoxic gene expression in ECs *in vitro*. **(A)** *SP1* mRNA levels in HUVECs after 24 h of hypoxia were detected *via* qPCR (*n* = 4). **(B)** SP1 protein levels in HUVECs after 24 h after siRNA transfection were detected by Western blot (*n* = 3). For each sample, 20 μg of protein were loaded onto polyacrylamide gels. Chemiluminescence with an exposure time of 390 s (SP1) or 120 s (GAPDH). **(C)** mRNA levels of genes, which were upregulated under hypoxia, were detected using qPCR after siRNA-mediated *SP1*-knockdown in combination with hypoxia (*n* = 4). **(D)** mRNA levels of genes, which were downregulated under hypoxia, were detected using qPCR after siRNA-mediated *SP1*-knockdown in combination with hypoxia (*n* = 4). Each dot resembles the mean value of all technical replicates from an independent experiment. *p ≤ 0.05, ^**^p ≤ 0.01, ^***^p ≤ 0.001.

Next, we aimed to investigate the effect of TF modulation on hypoxia-induced endothelial gene transcription. Silencing of endogenous SP1 with a specific siRNA resulted in a robust knockdown of SP1 on protein but not on mRNA level (Figures 2A,B). To examine whether SP1 could influence expression of selected hypoxia-sensitive genes in ECs, we quantified relative mRNA levels after siRNA application during hypoxic conditions *in vitro*. We observed that six out of eight genes with a suspected SP1 binding site within their upstream regulatory elements show altered mRNA levels after SP1 knockdown under hypoxia (Figures 2C,D).

For four (*ANPTL4*, *DNAH11*, *INHBA*, and *BGN*) of these five upregulated genes with a predicted binding site the hypoxia-driven upregulation was augmented after *SP1*-knockdown, even though the knockdown did not affect expression of these mRNAs under normoxic conditions, indicating a selective mechanism of SP1 between normoxia and hypoxia (Figure 2C). In contrast, out of the three downregulated genes with a predicted binding site, two (*LRP8* and *MCM4*) of these were shown to be unaffected on mRNA levels after *SP1* repression in hypoxic environment. Transcriptomic repression as a result of *SP1*-knockdown at normoxic conditions was abolished in hypoxic HUVECs (Figure 2D). In conclusion, SP1 shows a bilateral function under hypoxic signaling where it can either repress the upregulation of hypoxia-sensitive genes under hypoxic conditions or induce the gene expression under normoxic conditions. The latter only seems to occur if respective genes are downregulated as a result of hypoxic signaling. Combining these observations with our finding that *SP1* mRNA levels remain unchanged after 24 h of hypoxia in ECs (Figure 1D), regulation of hypoxic gene expression *via* SP1 appears to be mediated *via* modulation of its activity but not its quantity. Collectively, we hypothesize that SP1 activity is altered in human ECs under hypoxemia as transcriptomic regulation patterns after SP1-knockdown differ between hypoxia and normoxia. We observed SP1-mediated regulation in both conditions, thus we claim that not only SP1 but on top chromatin dynamics and hence, accessibility of binding sites possess a major role in the regulation of SP1-driven transcription in ECs under hypoxia.

**FIGURE 3:**
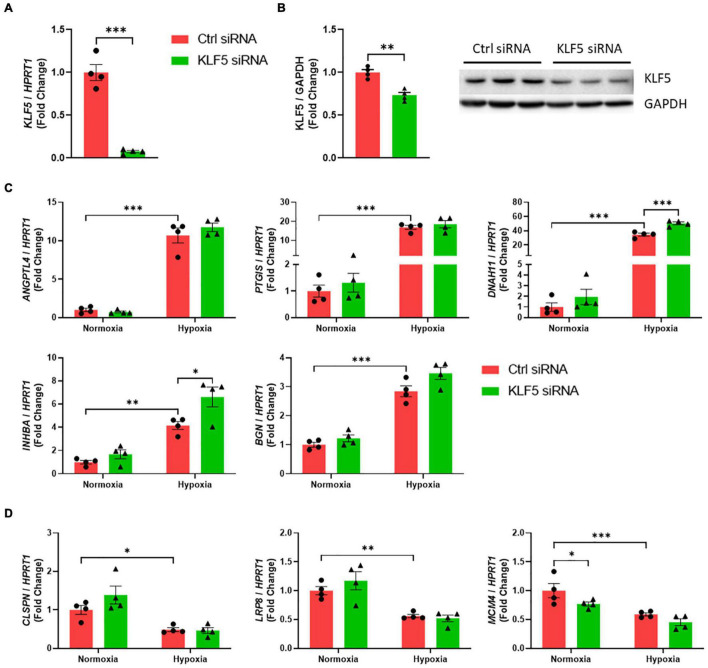
KLF5 does not regulate hypoxic gene expression in ECs. **(A)** *KLF5* mRNA levels in HUVECs after 24 h of hypoxia were detected *via* qPCR (*n* = 4). **(B)** KLF5 protein levels in HUVECs after 24 h after siRNA transfection were detected by Western blot (*n* = 4). For each sample, 30 μg of protein were loaded onto polyacrylamide gels. Chemiluminescence with an exposure time of 398 s (KLF5) or 13 s (GAPDH). **(C)** mRNA levels of genes, which were upregulated under hypoxia, were detected using qPCR after siRNA-mediated *KLF5*-knockdown in combination with hypoxia (*n* = 4). **(D)** mRNA levels of genes, which were downregulated under hypoxia, were detected using qPCR after siRNA-mediated *KLF5*-knockdown in combination with hypoxia (*n* = 4). Each dot resembles the mean value of all technical replicates from an independent experiment. *p ≤ 0.05, ^**^p ≤ 0.01, ^***^p ≤ 0.001.

In parallel, our proof of concept experiments revealed that *KLF5* mRNA levels were successfully downregulated by siRNA after 24 h (Figure 3A). In line, Western blot experiments confirmed the knockdown of KLF5 also on protein level (Figure 3B). Assessing mRNA levels of preselected genes after a combinatory treatment of hypoxia and siRNA transfection, we observed that *KLF5*-knockdown was only able to augment *DNAH11* and *INHBA* mRNA levels under hypoxia, whereas mRNA levels of the other predicted genes remain unchanged (Figures 3C,D). But similar to SP1, a *KLF5*-knockdown only affected *DNAH11* and *INHBA* mRNA levels under hypoxic conditions and not under regular culture conditions (Figures 2C, 3C), further strengthening our hypothesis of a selective regulatory mechanism induced by either a normoxic or hypoxic environment.

### SP1 Induces Inflammatory Response and Angiogenesis of Endothelial Cells

**FIGURE 4:**
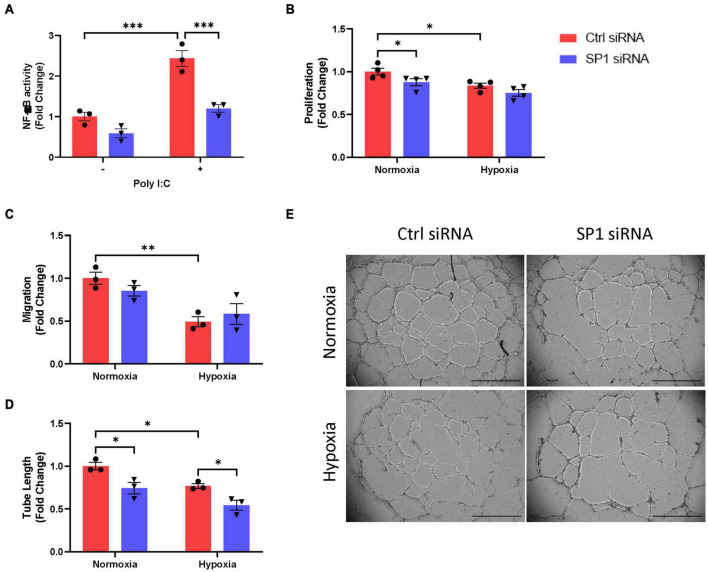
SP1 inhibits endothelial function. **(A)** Luciferase reporter assay was used to determine NF-κB signaling in HEK293FT cells after siRNA-mediated knockdown of *SP1* (*n* = 3). **(B)** Proliferation of HUVECs after *SP1*-knockdown and hypoxia was determined by BrdU-incorporation (*n* = 4). **(C)** Migration of HUVECs after *SP1*-knockdown and hypoxia was determined by scratch wound healing assay (*n* = 3). **(D)** Tube formation of HUVECs after *SP1*-knockdown and hypoxia was determined by tube formation assay (*n* = 3). **(E)** Representative images of tube formation assay in HUVECs after *SP1*-knockdown and hypoxia. Scale bar, 1,000 μm. Each dot resembles the mean value of all technical replicates from an independent experiment. *p ≤ 0.05, ^**^p ≤ 0.01, ^***^p ≤ 0.001.

Besides the control of hypoxic gene expression, we were wondering if KLF5 and SP1 actions also affect EC function. As stated earlier, low oxygen supply and inflammation are interconnected by HIF-1α and NF-κB (18, 19). Using a Luciferase-based reporter assay, we investigated if TFs could not only influence hypoxic gene transcription but also inflammatory response. Applying this construct, we were able to monitor NF-κB promoter activity dependent on pro-inflammatory conditions. By stimulating HEK293FT cells with a pro-inflammatory stimulus in terms of Poly I:C we observed an enhanced NF-κB signaling. The increased inflammatory response was attenuated after knockdown of both TFs, SP1 (Figure 4A) and KLF5 (Supplementary Figure 2A). A hallmark of ECs is the initiation of angiogenesis. Therefore, we tested whether siRNA-mediated knockdown of *SP1* and *KLF5* could interfere with angiogenic attributes of HUVECs such as proliferation, migration and tube formation under hypoxic conditions. By measuring the integration of BrdU into the DNA of dividing HUVECs, *SP1*-knockdown cells – similar to hypoxic ECs – exhibit decreased proliferation under normoxia, but not under hypoxia (Figure 4B), whereas *KLF5*-knockdown did not affect EC proliferation at all (Supplementary Figure 2B). This pattern appears to be similar to how SP1 seems to control hypoxic gene expression (Figure 2C). As opposed to this, EC migration was not affected by neither *SP1*- (Figure 4C) nor *KLF5*-knockdown *in vitro* (Supplementary Figure 2C). Eventually, both, EC proliferation and migration result in the formation of new capillary structures. By culturing HUVECs on a three-dimensional matrix, hypoxic ECs exhibit less tube formation compared to normoxic ECs (Figures 4D,E). A knockdown of *SP1* mRNA levels resulted in reduced tube formation after normoxia as well as after hypoxia (Figures 4D,E). Similar to EC proliferation and migration a KLF5-knockdown had no effect on EC tube formation, neither under normoxia nor hypoxia (Supplementary Figures 2D,E). In conclusion, SP1 might act as a mediator of inflammatory response *via* stimulating NF-κB signaling as well as angiogenic signaling in ECs by initiating EC proliferation and tube formation.

**FIGURE 5:**
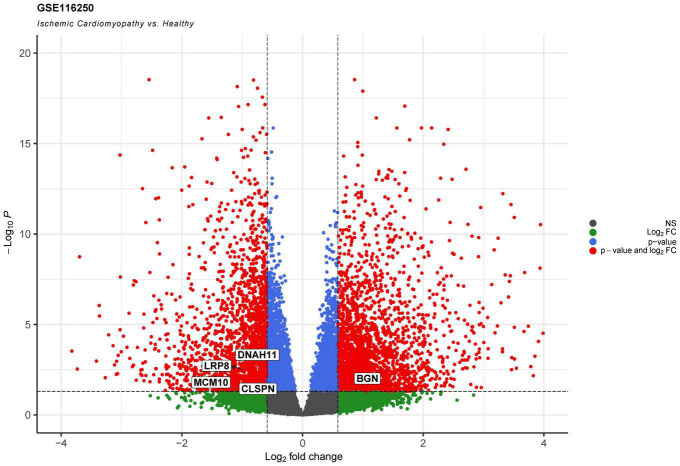
Volcano plot showing results of differential gene expression analysis of human heart tissue from ischemic heart failure in comparison with healthy controls. Red dots show significantly deregulated genes with logFC > | 0.585| and adjusted *p*-value < 0.05. Genes of interest are labeled.

In addition we analyzed a RNA-Seq data set (GSE116250) of human left ventricular tissue from donors with ischemic cardiomyopathy. Five out of the ten genes of interest were also significantly deregulated between ischemic heart tissue and non-failing hearts (Figure 5).

## Discussion

The epigenetic landscape of hypoxia is complex (20, 21). Multiple TFs such as HIF-1α and EPAS1 have a dynamic functional pattern in orchestrating gene expression in hypoxia. However, the interaction of these pioneering TFs with the existing epigenetic architecture may be key for the fine-tuning of biological outcomes. Our bioinformatics screening identified SP1 and KLF5 binding sites as common features of deregulated genes in a dataset from hypoxic HUVECs (8), indicating a broad supporting involvement of these TFs as intermediaries in oxygen-deficient conditions. Indeed, the knockdown of SP1 and KLF5 led to a strong de-repression of multiple predicted hypoxic factors, implying their roles as likely intermediaries in the process.

This study reports the key contribution of endothelial TFs SP1 and KLF5 during human EC hypoxic gene response. To our knowledge, this is the first study linking *in silico* and outcome analysis for identified TFs underlining the importance of herein presented results. Despite a potential false positive rate of putative promoter binding predictions, we were able to identify SP1 and KLF5 as highly relevant biological factors and model their role within the epigenetic regulation of hypoxia. SP1 and KLF5 were indeed independently found in other studies as major players in low-oxygen conditions (22–26). Therefore our *in silico* analysis approach depicts a very efficient way to narrow down a broad range of results coming from high throughput experiments such as RNA-sequencing to gain precise insights into the regulatory biology behind a certain setting. This allows systematic planning of validation experiments underlining a hypothesis without cost- and time-intensive experiments such as ChiP-Seq. Comparing our approach with other available methods (27, 28) it is noteworthy, that our method is meant to be an upstream step for experimental validation not a stand-alone method. Also we focus more on identifying major regulatory key molecules than discovery of novel binding motifs. The library used in our approach (TFBStools) also contains a wrapper function for a MEME analysis, which, in line with other tools, focuses more on “*de novo*” discovery of motifs. We preferred to establish a method to efficiently deliver an overview over regulatory effects in a NGS data set of interest. In the method proposed by Srivastava et al. (29), for example, the user needs to create and compile sequence files of the regions of interest as well as manually select the TFs. Our approach combines all steps in an easy to use R script where users only need to enter the organism of interest and a list of genes with identifiers as comma separated text file.

Since physiological processes such as angiogenesis and inflammation take part in multiple disease models, there is an interest in developing drugs that modulate SP1 signaling in ECs under hypoxia. Thus, investigating the modulation of KLF5 and SP1 signaling could give new insights into the treatment of various disease models. Targeting highly interconnected intermediaries such as SP1 and KLF5 may have clinical relevance. In the context of recovery from myocardial ischemia, hypoxia was reported to have both beneficial and detrimental effects (22). In line, clinical translation of our findings to a dataset of human ischemic heart failure samples (GSE116250) revealed significant deregulation of five endothelial-cell related genes (Figure 5). Speculatively, the positive aspects could be directly extracted on a molecular level by maintaining SP1 or KLF5 levels, as SP1 supported the angiogenic TNF-α thymidine phosphorylase pathway (23) and VEGF (24). On the other hand, SP1 (25) and KLF5 inhibition slowed angiogenesis (26), which may be a useful strategy in oncotherapy. Moreover, we previously showed that modular SP1 and EN1 transcriptional binding mediates pro-fibrotic effects of TGFβ signaling, in which EN1 knockout reverses experimental skin fibrosis (30). Our study showed impaired proliferation, migration, and consequently tube formation in hypoxic ECs, which furthermore was intensified after TF knockdown. This is contrast to *in vivo* observations where hypoxia induces aforementioned features. These findings originate in the different environmental conditions between *in vitro* and *in vivo* studies. ECs *in vivo* are predominantly quiescent before they become activated by hypoxic sensing and subsequently initiate angiogenesis *via* proliferation and migration. *In vitro*, ECs are constantly activated which is underlined by their constant proliferation.

In summary, we have developed an intuitive *in silico* approach to screen for TF regulation, allowing the modeling of significant biological processes including, but not limited to, hypoxia. A potential mediator by which SP1 affects EC function could be Activin A – an INHBA homodimer – and ANGPTL4, as these could already be shown to inhibit tube formation and proliferation of normoxic as well as hypoxic ECs (31–34). This could explain the attenuated tube formation ability and proliferation of ECs after SP1-knockdown.

## Materials and Methods

### *In silico* Prediction of Transcription Factor Binding Elements

The whole TF-analysis is presented as an easy-to-use script for the R programming language. Except for initially setting organism of choice; no advanced programming skill are necessary and the script runs automatically. All user inputs are described as comments within the R-script. After termination, CSV-files containing the results from promoter screening as well as the actual gene-TF prediction are saved to the current working directory. The session info is also saved as a txt-file.

The computation was performed on a Windows 10 machine with Intel i5 CPU and 32 GB memory, but is expected to also work on smaller systems with a longer but reasonable timespan for computation. Analysis follows our previously applied promoter profiling method (30). Upstream (−800 bp from TSS) sequences for genes of interest were retrieved using the bioMart package (v 2.48.3) in R (v. 4.1.1). Position weight matrices for human TFs were retrieved using JASPAR2018 package (v 1.1.1). Sequence scanning was performed using *searchSequence* function provided by TFBStools package (v. 1.30.0). All resulting hits were ranked by calculated binding score in a decreasing order and the top one percent was kept. Using the built-in function *TFMPvalue*, *p*-values for the related binding scores are calculated. Since we select only scores within the 1%-quantile, *p*-values are always significant, but can be stored for further inspection. Multiple hits for a TF-gene combination were eliminated. Unique hits were counted and divided by the number of overall genes of interest to obtain the enrichment ratio.

### RNA-Seq Analysis of Human Left Ventricle Tissue

Raw reads were obtained from NCBI Sequence Read Archive (SRA) using fastq-dump function implemented in sratoolkit.^1^ Alignment to reference genome (GRCh38) and read mapping was performed using Rsubread (35). Differential gene expression was performed using our previously published tool tRomics (36). Genes with logFC > | 0.585| and adjusted *p*-value < 0.05 were considered differentially expressed.

### Cell Culture and Transfection

HUVECs (Lonza, Switzerland) were cultured in EBM™-2 Endothelial Cell Growth Basal Medium-2 (Lonza, Switzerland), supplemented with Hydrocortisone, hFGF-B, VEGF, R3-IGF-1, Ascorbic Acid, hEGF, GA-1000 (Lonza, Switzerland) and 10% FBS (Gibco, Ireland). The cells were incubated in a humidified incubator (Binder, Germany) at 37°C and 5% CO_2_. For hypoxia studies, HUVECs were incubated at 0.2% O_2_ for 24 h 1 day after seeding the cells. Transfection of siRNAs (Table 1) was performed 1 day after seeding the cells. The siRNAs were diluted in Opti-MEM™ I Reduced Serum Medium (Gibco, Ireland) to a final concentration of 10 μM. Separately, Lipofectamine 2000 (Invitrogen, United States) was diluted 1:125 in Opti-MEM™ I Reduced Serum Medium. Both mixes were combined after an incubation time of 5 min at room temperature and incubated for another 20 min at room temperature. HUVECs were incubated in the transfection mix for 4 h at 37°C and 5% CO_2_. Afterward, medium was changed to regular culture medium and HUVECs were cultured for 24 h either at normal (21% O_2_) or hypoxic conditions (0.2% O_2_). All experiments were performed in passage five to nine cells.

**TABLE 1:** siRNAs for transfection.

Name	Company	Catalog #
Control siRNA-A	Santa Cruz	sc-37007
KLF5 siRNA	Santa Cruz	sc-37718
SP1 siRNA	Santa Cruz	sc-29487

HEK293FT were cultured in DMEM, high Glucose (Gibco, Ireland), supplemented with 10% FBS (Gibco, Ireland) and 1% Penicillin/Streptomycin (Gibco, Ireland). For plasmid transfection, 100 ng of DNA was diluted in Opti-MEM™ I Reduced Serum Medium. In parallel, Lipofectamine 2000 was diluted 1:125 in Opti-MEM™ I Reduced Serum Medium and combined with the diluted DNA after 5 min incubation time at room temperature. The mix was incubated for further 20 min at room temperature and added to the cells afterward. After 4 h incubation time at 37°C and 5% CO_2_, medium was changed.

### RNA Isolation and Quantitative Real-Time PCR

RNA was isolated *via* QIAzol Lysis Reagent (Qiagen, Germany) according to the manufacturers instructions. Subsequently, 600–1,000 ng of RNA were transcribed using an Oligo-dT cDNA synthesis protocol (Bio-Rad, United States). For each qPCR reaction 20 ng cDNA were used and mRNA levels were assessed by using the ABsolute™ Blue QPCR Mix, SYBR Green, Low ROX (Thermo Fisher, United States) in a QuantStudio™ 7 Flex Real-Time PCR system (Applied Biosystems, United States). Data were analyzed *via* standard curve method and mRNA levels of every gene were normalized to the relative levels of *HPRT1* mRNA. All primers used in these experiments are listed in Table 2 [*INHBA* (QT00201586) and *KLF5* (QT00074676) primers were purchased from Qiagen (Germany)].

**TABLE 2:** qPCR primer sequences.

Target	Fwd primer sequence (5′→3′)	Rev primer sequence (5′→3′)
*ANGPTL4*	TCCTGGACCACAAGCACCTA	ATCGTGGCGCCTCTGAATTA
*BGN*	GGACACACCGGACAGATAGA	CATCATGAATGGCCCATCGTC
*CLSPN*	GGTGAAGGCCCCCAAAATCC	AACGCTGCTTCAAGGCTTCC
*DNAH11*	CAGTATTTGGAAAGCGAGGACA	AGTTTATGGTTTGCATCTCTTGGA
*FEN1*	TGAGAAGGGAGAGCGAGCTTA	GCCAGGCCTTGAATTCCCAT
*HPRT1*	AGGACTGAACGTCTTGCTCG	GTCCCCTGTTGACTGGTCATT
*LRP8*	GATGAGTCCGAGGCCACTTG	GACACTCGTTCAGCCAGGTA
*MCM4*	GACATGGCGGTGCTAAAGGA	GCTGTCGAGGGTATGCAGAA
*MCM10*	TCCCAACCCCTACAGACGAT	ATCAGTTTTCGGCCGGTCAT
*PTGIS*	TGGAGAGTTACCTGCTGCAC	TGTGGGAGAGTGGTCGTCT
*SP1*	AGGCGAGAGGCCATTTATGT	TCTTCTCACCTGTGTGTGTACG

### Protein Isolation and Western Blot

Protein extraction and Western blotting was performed similar to our previous work (37). Cells were washed with PBS, centrifuged, resuspended in cell lysis buffer (Bio-Rad, United States) and sonicated by a Bioruptor^®^ Pico sonication device (Diagenode, Belgium). From each sample either 20 or 30 μg of protein were loaded onto polyacrylamide gels for electrophoretic band separation and transferred to a nitrocellulose membrane (Bio-Rad, United States). Blocking of membranes was performed by incubation for 1 h in 5% blocking milk solution. The membranes were incubated together with the primary antibody overnight at 4°C on a rotator. After rinsing, the membranes were exposed to secondary IgG-HRP antibodies, ultimately being washed again and visualized with luminol-containing substrate solution or Clarity Western ECL Substrate (Bio-Rad, United States). Protein levels were normalized to GAPDH protein levels. Precision Plus Protein™ WesternC™ Blotting Standard (Bio-Rad) was utilized as a protein standard. Antibodies used for detection can be found in Table 3.

**TABLE 3:** Antibodies for Western blot experiments.

Antigen	Company	Catalog #	Origin	Working dilution
GAPDH	Abcam	ab8245	Mouse	1:30,000
KLF5	Abcam	ab24331	Rabbit	1:500
SP1	Abcam	ab13370	Rabbit	1:2,500
Anti-mouse-HRP	Cell Signaling	7076	Horse	1:10,000
Anti-rabbit-HRP	Cell Signaling	7074	Goat	1:10,000

### Luciferase Reporter Assay

A Luciferase Reporter Assay in HEK293FT cells was used to determine the activity of inflammatory NF-κB signaling as previously described (38). In a 48-well format, 175,000 HEK293FT cells were co-transfected with the pSGNluc plasmid, a β-galactosidase control plasmid (Promega, United States) and respective siRNA as described earlier. The pSGNluc plasmid contains multiple NF-κB binding sites and therefore, enables evaluating NF-κB activity *via* detection of Luciferase activity. After transfection, medium was changed to serum-free medium containing 300 ng/ml Poly I:C incubated on HEK293FT cells for 24 h at 37°C and 5% CO_2_. Luminescence was detected using a Synergy HT Multi-Detection Microplate Reader (BioTek, United States) and in the end, Luciferase activity was normalized to β-galactosidase activity.

### Tube Formation Assay

Wells of a 96-well plate were coated with 40 μl Matrigel Basement Membrane Matrix, LDEV-free (Corning, United States) and incubated for 30 min at 37°C prior to seeding the cells. At the end of the treatment, 10,000 HUVECs were transferred onto the Matrigel Matrix and incubated at 37°C and 5% CO_2_. Brightfield images were captured 24 h after seeding using a Cytation 1 Cell Imaging Multi-Mode Reader (BioTek, United States). Total tube length was determined with ImageJ by the Angiogenesis Analyzer tool (39).

### Scratch Wound Healing Assay

A two-dimensional Scratch Wound Healing Assay was used to determine migration ability of HUVECs. In a 96-well plate, 30,000 HUVECs were seeded per well and treated on the next day as described. After the treatment, cells were stained with 5 μg/ml Hoechst 33342 (Thermo Fisher, United States) for 15 min at 37°C and 5% CO_2_. Next, a wound was scratched into the cell layer using a 20 μl pipette tip (Sarstedt, Germany). To remove dead cells, the plate was washed once with PBS and fresh medium was added. Subsequently, images were captured every 2 h for 16 h using a Cytation 1 Cell Imaging Multi-Mode Reader (BioTek, United States) with the first images being taken directly at the start. For the duration of the imaging process, the cells were incubated in a BioSpa 8 Automated Incubator (BioTek, United States) at 37°C, 21% O_2_ and 5% CO_2_. Migration was analyzed with ImageJ by measuring the covered area for every timepoint and calculating the migration index.

### Proliferation Assay

Proliferation of HUVECs was assessed by Cell Proliferation ELISA, BrdU (colorimetric) (Roche, Switzerland). A total of 5,000 HUVECs were seeded in a 96-well plate 1 day prior to the transfection and hypoxia treatment. After 24 h incubation at either normoxic or hypoxic conditions, medium was changed to medium containing BrdU (1:1,000) and incubated for further 24 h at 37°C, 21% O_2_ and 5% CO_2_. After the incubation time, the assay was performed according to the manufacturers manual and absorbance was measured using a Synergy HT Multi-Detection Microplate Reader (BioTek, United States).

### Statistics

All statistics were performed with GraphPad Prism (v 8.3.1) (GraphPad, United States). Statistical significance between two groups was determined either by Student’s *t*-test or Welch’s *t*-test. Statistical significance among three or more groups was determined by ANOVA. All values are displayed as means ± SEM. Statistical significance was considered for *p* ≤ 0.05.

## Data Availability Statement

All data generated in this study are available in manuscript and as supplement online. The RNASeq datasets are available at GEO with accession numbers: GSE30775 and GSE116250. The R script is available at https://github.com/max-fuchs/tf_screen.

## Author Contributions

AS, MF, JF, TT, and MK: conceptualization. AS, MF, JF, and MK: methodology, investigation, and visualization. AS, SS, KS, JW, AJ, AP, and JF: experiment. MF, CL, JD, TD, and MK: software. AS, MF, CL, KS, MJ, KX, JF, and MK: formal analysis. TT and MK: resources and funding acquisition. AS, MF, CL, KX, and MK: data curation. AS and MK: writing—original draft and preparation. AS, MF, SS, MJ, JD, TD, JF, and TT: writing—review and editing. JF, TT, and MK: supervision. JF and MK: project administration. All authors have read and agreed to the final version of the manuscript.

## Conflict of Interest

TT has filed and licensed patents regarding non-coding RNAs in CVD. TT is founder and shareholder of Cardior Pharmaceuticals GmbH. The remaining authors declare that the research was conducted in the absence of any commercial or financial relationships that could be construed as a potential conflict of interest.

## Publisher’s Note

All claims expressed in this article are solely those of the authors and do not necessarily represent those of their affiliated organizations, or those of the publisher, the editors and the reviewers. Any product that may be evaluated in this article, or claim that may be made by its manufacturer, is not guaranteed or endorsed by the publisher.

## Footnotes

1
https://github.com/ncbi/sra-tools
