# Supplementary material for: Deciphering Pro-angiogenic Transcription Factor Profiles in Hypoxic Human Endothelial Cells by Combined Bioinformatics and in vitro Modeling
Source: Front Cardiovasc Med. 2022 Jun 17;9:877450. doi: 10.3389/fcvm.2022.877450 (PMC9247153; doi:10.3389/fcvm.2022.877450)
Supplement: Supplementary file 2 [file Data_Sheet_1.pdf]

Supplementary Material

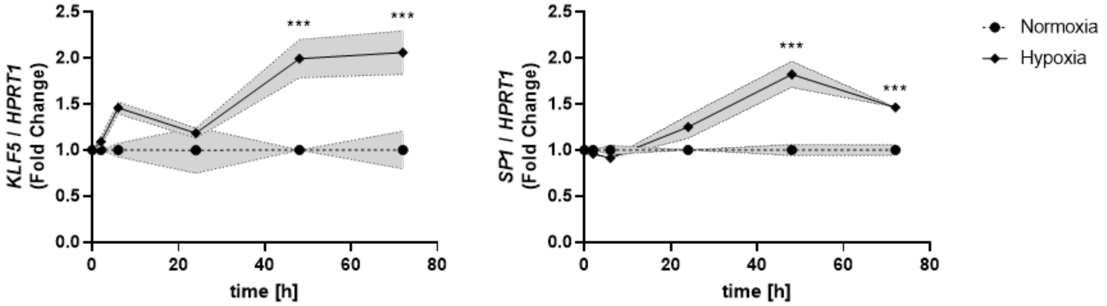

Supplementary Figure 1. Transcription factors SP1 and KLF5 are potential regulators of EC transcriptional response to hypoxic signaling. Timelapse qPCR data of *KLF5* and *SP1* mRNA levels in HUVECs after hypoxia ($n = 3$). ***: $p \leq 0.001$

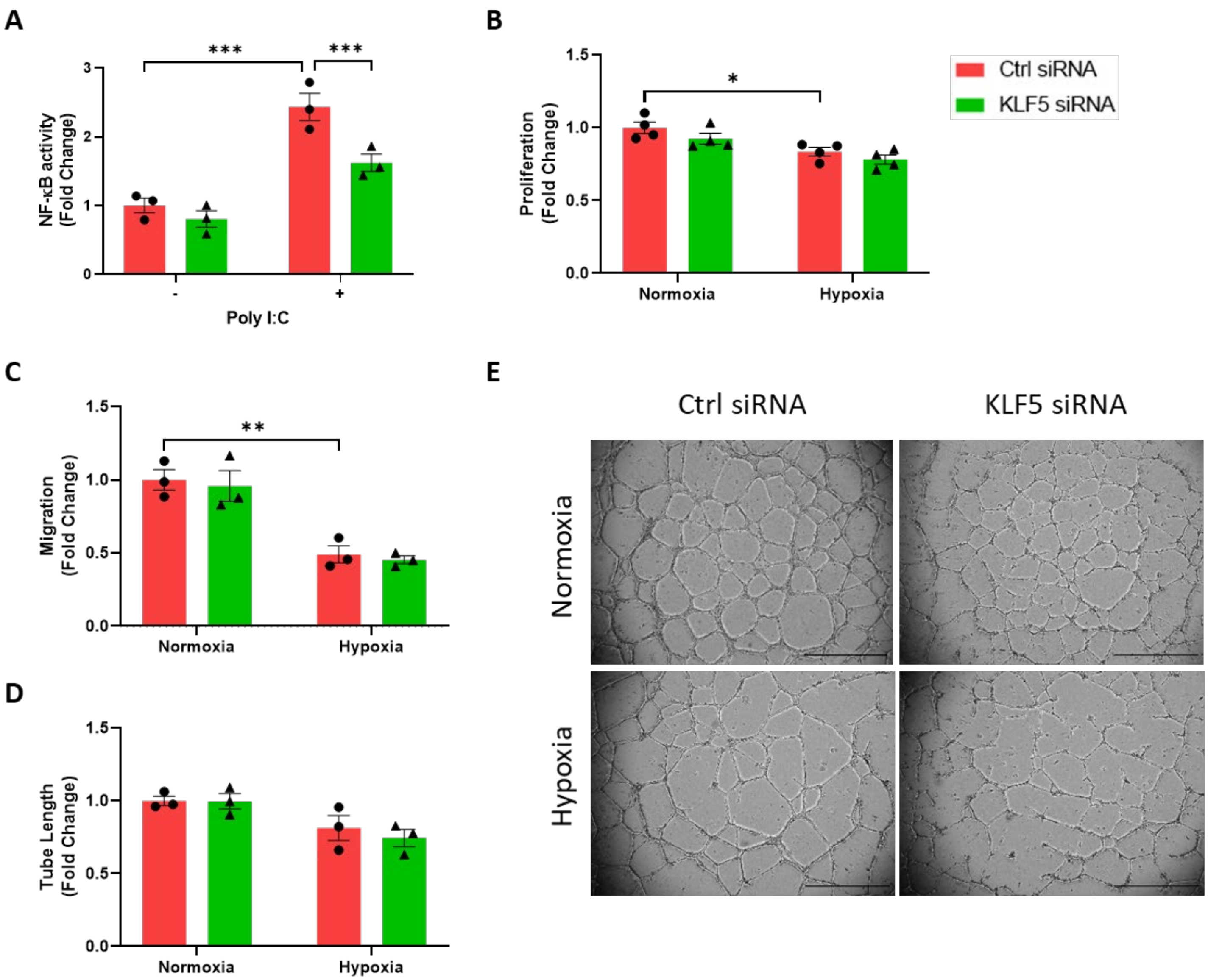

Supplementary Figure 2. KLF5 partially inhibits endothelial function under hypoxia. (A) Luciferase reporter assay was used to determine NF-κB signaling in HEK293FT cells after siRNA-mediated knockdown of *KLF5* ($n = 3$). (B) Proliferation of HUVECs after *KLF5*-knockdown and hypoxia was determined by BrdU-incorporation ($n = 3$). (C) Migration of HUVECs after *KLF5*-knockdown and hypoxia was determined by Scratch Wound Healing Assay ($n = 3$). (D) Tube Formation of HUVECs after *KLF5*-knockdown and hypoxia was determined by Tube Formation Assay ($n = 3$). (E) Representative Images of Tube Formation Assay in HUVECs after *KLF5*-knockdown and hypoxia. Scale Bar = 1000 µm. *: $0.01 < p \leq 0.05$, **: $0.001 < p \leq 0.01$, ***: $p \leq 0.001$
